# Can Smartphones Enhance Telephone-Based Cognitive Assessment (TBCA)?

**DOI:** 10.3390/ijerph10127110

**Published:** 2013-12-12

**Authors:** Rick Yiu-Cho Kwan, Claudia Kam-Yuk Lai

**Affiliations:** The School of Nursing, The Hong Kong Polytechnic University, Hung Hom, Kowloon, Hong Kong; E-Mail: rick.kwan@polyu.edu.hk

**Keywords:** telephone, cognitive assessment, smartphone, dementia

## Abstract

TBCA has emerged to solve the limitations of administering cognitive assessments face-to-face. The recent development of telephones and knowledge advances in the area of cognitive impairment may affect the development of TBCA. The purpose of this paper is to discuss how smartphones can be used to enhance the applicability of TBCA, which has previously been administered by conventional telephone. This paper will first review, describe and critique the existing TBCA instruments. It will then discuss the recent developments in tele-technology, the popularity of tele-technology among the elderly, potential benefits and challenges in using smartphones for cognitive assessment, and possible future developments in this technology. In the systematic review, eighteen TBCA instruments were identified. They were found to be valid in differentiating between people with and without dementia. TBCA was previously found to be launched on a conventional telephone platform. The advances in understanding of cognitive impairment may demand that telephones be equipped with more advanced features. Recently, the development and penetration of smartphones among the elderly has been rapid. This may allow the smartphone to enhance its TBCA applicability by overcoming the limitations of the conventional telephone, rendering the TBCA more efficient in addressing the increasing demand and complexity of cognitive assessments in the future. However, more research and technology developments are needed before smartphones can become a valid platform for TBCA.

## 1. Introduction

Ageing is a global problem, with the total number of older people expected to double in the next 25 years, from 606 million in 2000 to over 1.2 billion by 2025 [1]. The prevalence of dementia and cognitive impairment increases exponentially with age [2,3,4]. By 2050, the prevalence of dementia is expected to quadruple, so that one in 85 people will be living with dementia [5]. The rapid expansion of the number of elderly as well as younger people with cognitive impairment or dementia demands an increase in cognitive assessment services. 

Cognition is a core function of the cerebrum. It includes memory, learning, language, visuo-spatial function, attention resources, mental speed and executive function [6]. When cognition is impaired (*i.e.*, cognitive impairment), a person has trouble with the aforementioned processes, which begins to affect the things he or she can do in everyday life. There are many possible reasons for the decline of cognitive function, such as Alzheimer’s disease and stroke. The degree of cognitive impairment ranges from mild to severe grade [7]. When the cognitive impairment is severe enough to interfere with daily function, the condition is called dementia [8]. In the last decade, the understanding of cognitive impairment has advanced. The cutoff between dementia and non-dementia has become more complex with increased understanding of the prodromal stage of dementia, such as mild cognitive impairment (MCI) [9] and cognitive impairment not dementia (CIND) [10]. The problem lies in determining at what stage the person will progress from the prodromal stage to full-fledged dementia (as indicated by a cut-off score). These advances in understanding cognitive impairment and dementia make the assessment more complicated than in the past.

Despite technological advances in the use of medical bio-chemistry and nuclear imaging in dementia diagnosis, the role of clinical assessments (e.g., neuropsychological or cognitive assessment) still cannot be replaced, because they provide flexibility to general healthcare providers who may not have the technological tools available [11]. Cognitive assessment is traditionally administered face-to-face. The assessor in the face-to-face cognitive assessment may ask the client or proxy to answer some questions or perform some cognitive tasks in order to identify the client’s cognitive status. Comprehensive cognitive assessment administered face-to-face is the “gold standard” because its reliability and diagnostic accuracy are high. However, the usefulness of face-to-face cognitive assessment is limited by many factors, such as long testing time and transportation for the elderly who may have mobility and financial difficulties [12]. Telephone-based cognitive assessment (TBCA) has therefore emerged to solve the limitations. TBCA can be conducted anywhere that a telephone is available, and it can be completed in a shorter period of time [13]. 

In the last three decades, many cognitive assessment tools have been validated to be administered over the telephone. Two recent studies reviewed some cognitive assessment instruments used via telephone [14,15], identifying many commonly-used TBCA instruments and showing that they were valid in differentiating between people with and without dementia. However, the review papers [14,15] did not discuss the characteristics of the instruments, such as the cognitive domains to be tested and the limitations of the tools. Healthcare services can be improved by technology [16]. With regard to the recent development of tele-technology, such as the proliferation of smartphones, it is important to re-appraise the existing TBCA tools in order to examine how technology can further upgrade the TBCA in order for cognitive assessment to better serve its purposes.

The purpose of this paper is to discuss how smartphones can be used to enhance the applicability of the TBCA, which has previously been administered by conventional telephone. This paper will first review, describe and critique the existing TBCA instruments. It will then discuss recent developments in tele-technology, the popularity of tele-technology among the elderly, potential benefits and challenges of using smartphones for cognitive assessment, and the possible future development of this technology.

## 2. Methods

In a review of the existing TBCA instruments, a systematic review was performed to identify previously validated TBCA instruments. The databases searched included the Ovid MEDLINE, Health and Psychosocial Instruments, CINAHL and PsycInfo. The search period was from 1980 to 2013. The keywords included “telephone”, “cognitive assessment” and “dementia”. After limiting the findings to those articles with abstracts and in English only, 454 papers were identified. All the abstracts were reviewed to see whether they fulfilled the inclusion criteria of this review. For the inclusion criteria, only papers examining the validity and reliability of a TBCA were selected. Finally, eighteen papers were identified that examined eighteen different TBCA instruments.

## 3. Results

This systematic review will describe the characteristics of the eighteen TBCA instruments in two ways: the modes of administration and their validity.

### 3.1. The Modes of Administration

The eighteen TBCA instruments can be categorized into three modes of administration: subject-based test, informant-based test, and combined test (Table 1).

#### 3.1.1. Subject-Based Tests

Subject-ased tests refer to assessments that focus mainly on the performance of the subjects in designated cognitive tasks during the test. They are mainly adopted from the face-to-face instruments for cognitive assessment. The Mini-Mental Status Exam (MMSE) [17] is the most common prototype to be modified. The MMSE mainly assesses five cognitive domains: orientation, attention and calculation, memory, language, and visual construction. It demands visual communication and writing tasks, which cannot be administered using the conventional telephone. To solve this problem, it was adopted as a telephone-based instrument by eliminating those items that could not be administered via the conventional telephone (e.g., as the Telephone-Assessed Mental State, TAMS) [18]. However, eliminating some assessment items without considerable compensation compromised its usefulness in assessing multiple cognitive domains.

**Table 1:** Cognitive assessment tools administered via telephone (N = 18).

Instrument	Author	Year	Reference
*Subject-Based Tests*			
Mitsis’ Battery	Mitsis *et al*.	2009	[12]
Telephone Assessed Mental State (TAMS)	Lanksa *et al*.	1993	[18]
Telephone Interview for Cognitive Status (TICS)	Brandt *et al*.	1988	[19]
Telephone Interview for Cognitive Status—Modified (TICS-m)	Welsh *et al*.	1995	[20]
Adult Lifestyles and Function Interview—Mini Mental Status Exam (ALFI-MMSE)	Roccaforte *et al*.	1992	[21]
Telephone Screen Protocol (TELE)	Gatz *et al*.	1995	[22]
Brief Test of Adult Cognition by Telephone (BTACT)	Tun & Lachman	2006	[23]
Telephone Cognitive Assessment Battery (TCAB)	Debanne *et al*.	1997	[24]
Cognitive Assessment of Later Life Status (CALLS)	Crooks *et al*.	2007	[25]
Cognitive Telephone Screening Instrument (COGTEL)	Kliegel *et al*.	2007	[26]
Rankin’s Battery	Rankin *et al*.	2004	[27]
Taichman’s Battery	Taichman *et al*.	2005	[28]
Rapp’s Battery	Rapp *et al*.	2012	[29]
*Informant-Based Test*			
Telephone Dementia Questionnaire (TDQ)	Kawas *et al*.	1994	[30]
Informant Questionnaire on Cognitive Decline in the Elderly (IQCODE)	Jorm *et al*.	1996	[31]
Symptoms of Dementia Screener (SDS)	Mundt *et al*.	2000	[32]
*Combined Test*			
Structured Telephone Interview for the Dementia Assessment (STIDA)	Go *et al*.	1997	[33]
Brief Screen for Cognitive Impairment (BSCI)	Hill *et al*.	2005	[34]

Efforts have been made by others to modify some telephone-unfriendly items. The following instruments are based on MMSE but modified the telephone-unfriendly items: the Telephone Interview for Cognitive Status, TICS; the Telephone Interview for Cognitive Status—modified, TICS-m; and the Adult Lifestyles and Function Interview—Mini Mental Status Exam, ALFI-MMSE [19,20,21]. For example, the ALFI-MMSE modified the “naming” task of the MMSE by asking “what is the thing called that you are speaking into?” over the phone. Rather than directly modifying the MMSE, some other scientists designed a new instrument or combined several existing cognitive assessment instruments in an attempt to include more cognitive domains and spare the telephone-unfriendly items. Instruments using this strategy include: the Telephone Screen Protocol, TELE; the Brief Test of Adult Cognition by Telephone, BTACT; the Telephone Cognitive Assessment Battery, TCAB; the Cognitive Assessment of Later Life Status, CALLS; the Cognitive Telephone Screening Instrument, COGTEL; Rankin’s battery; Mitsis’s battery; Taichman’s battery; and Rapp’s battery) [12,22,23,24,25,26,27,28,29]. For example, COGTEL uses six subtests that cover important domains of cognitive function: verbal short-term memory, verbal long-term memory, working memory, verbal fluency, inductive reasoning, and prospective memory. All the subtests are telephone-friendly because they do not require visual communication or writing tasks. 

The similarities among the subject-based TBCA instruments are that they all assess multiple cognitive domains, and the telephone-unfriendly items are eliminated. The main differences are that different instruments may cover different cognitive domains, and they use different strategies to modify the telephone-unfriendly items. The subject-based tests rely on the subject’s clinical performance, which closely reflects the subject’s cognitive function. When these tests are administered via telephone, some cognitive domains cannot be assessed technically, such as visual construction. Also, without visual communication, it is more difficult for subjects with possible cognitive and hearing impairment to comprehend the instructions. These factors may post threats to the applicability of subject-based tests.

#### 3.1.2. Informant-based Tests

Informant-based tests refer to assessments that are designed to be responded to by others who know about the subject. They require only that the subjects’ caregivers report their observations on the activity and functioning level of the subjects. In this case, the informant can be the caregiver, either formal or informal, of the subject. The informant-based instruments basically adopt face-to-face testing, even for TBCA instruments (e.g., the Telephone Dementia Interview, TDS; the Informant Questionnaire on Cognitive Decline in the Elderly, IQCODE; and the Symptoms of Dementia Screener, SDS) [30,31,32]. Unlike the subject-based tests, modification is generally not needed because the respondent is not hindered in his or her response as a result of his or her cognitive ability. It provides an alternative when some participants are unable to be assessed over the phone for various reasons, such as deafness or disease. The validity of this instrument depends heavily on the availability of caregivers and the depth of their knowledge about the subject’s functioning level.

However, administration of the informant-based tests via telephone does not significantly resolve the problems of the face-to-face versions. The caregivers still have to talk simultaneously with the assessors to complete the test. If the assessors and the caregivers are not simultaneously available, the availability problem is not solved. It also does not resolve the problem that arises when caregivers have an inadequate understanding of the subject’s functioning level.

#### 3.1.3. Combined Tests

Combined tests refer to assessments that use both subject- and informant-based tests. Both the subject and the informant are assessed. This method aims to balance the accuracy and feasibility of the two types of test. Some face-to-face instruments (e.g., the Structured Telephone Interview for the Dementia Assessment, STIDA, and the Brief Screen for Cognitive Impairment, BSCI) [33,34], which combined both subject- and informant-based tests, were adopted directly for TBCA. Modification was generally not needed because both the subject- and informant-based components had already been adapted to be administered via telephone, so that there were no telephone-unfriendly items. 

The emergence of the combined tests aimed at providing more information for the clinician about the subjects’ cognitive function. Technically, when the combined test is administered via telephone, it does not resolve the problems of the subject-based (e.g., no visual communication) and informant-based tests (e.g., availability of the caregivers).

### 3.2. The Validity

Most TBCA instruments have undergone certain validation procedures, although the rigor of the studies varies. The TBCA cited here were validated by several methods, namely, comparison with medical assessment by a physician, comparison with the same instrument administered face to face, and comparison with other validated instruments (Table 2). Some of the instruments have been examined for reliability.

Some TBCA instruments were compared with the medical assessment according to the clinical gold standard in order to examine the criterion validity. For example, the TICS [19] was validated by comparison with the medical assessment according to the National Institute of Neurological and Communicative Disorders and Stroke-Alzheimer’s Disease and Related Disorder Association (NINCDS-ADRDA) criteria for clinical diagnosis of probable Alzheimer’s disease. These validation studies showed that most of the instruments (TICS, TICS-m, TELE, TCAB, TDQ, IDCODE, SDS, STIDA) could accurately differentiate between people with and without dementia or cognitive impairment. The sensitivity of the tools validated in this approach ranged from 0.89 to 1.0, and the specificity from 0.83 to 0.97 [19,20,22,24,30,31,32,33]. 

Some TBCA instruments were compared with the face-to-face versions of the same instruments (e.g., ALFI-MMSE, IQCODE) in order to examine the criterion validity, as the face-to-face versions had previously been validated. Previous studies showed that many telephone-based versions correlated well with the face-to-face versions (r: 0.73-0.91) [21,26,31]. The evidence supported that they were also comparably valid when administered over the telephone.

Other TBCA instruments were compared with other forms of well-validated instruments (e.g., MMSE, ADAS-Cog, CDR) in order to examine the criterion validity. Some studies showed that the correlations between the TBCA instruments (e.g., ALFI-MMSE, TAMS & BSCI) and the well-validated instruments were good (r: 0.65–0.94) [18,21,34]. Another study showed that TBCA instruments (e.g., STIDA) accurately ranked the severity of dementia with weighted kappa of 0.81 [33]. Therefore, TBCA instruments are valid for dementia screening as well as staging the severity of dementia. Not all of the validation studies reported the reliability. The most common forms of reliability reported were test-retest reliability and inter-rater reliability. For example, the inter-rater reliability of the TBCA was good (kappa: 0.82–0.90) [24], and the test-retest reliabilities of the TICS (r = 0.97) [19] were also high. Other studies provided no such information about the TBCA instruments (e.g., TAMS, TELE, TDQ) [18,22,30].

In these validation studies, the TBCA were found to be valid in differentiating between people with and without dementia, and in staging the severity of the dementia. The performance of the TBCAs is highly comparable with that of many validated face-to-face cognitive assessment instruments. Many of the TBCAs were highly reliable. Therefore, TBCA is a valid method that deserves to be further developed and upgraded in order to serve its purposes better.

**Table 2:** Validity of cognitive assessment tools administered via telephone (N = 18).

Name	Year	Author	Comparison with Medical Assessment	Comparison with Face-to-Face	Comparison with Other Instruments	Score
*Subject-based Tests*						
Mitsis’s	2009	Mitsis *et al*. [12]		NSMD *		
TAMS	1993	Lanksa *et al*. [18]			ρ: 0.81 (c/w MMSE) ρ: −0.80 (c/w ADAS)	
TICS	1988	Brandt *et al.* [19]	Sen: 1.00, Spec: 0.83		r: 0.94 (c/w MMSE)	0–41
TICS-m	1995	Welsh *et al*. [20]	Sen: 1.00, Spec: 0.86			0–50
ALFI-MMSE	1992	Roccaforte *et al*. [21]		r: 0.85	r: 0.85 (c/w MMSE)	
TELE	1995	Gatz *et al*. [22]	Sen: 1.00, Spec: 0.91			
BTACT	2005	Tun & Lachman [ 23]		r: 0.56–0.95		
TCAB	1997	Debanne *et al*. [24]	Sen: 0.93, Spec: 0.97 (questionable) Sen: 0.98, Spec: 0.85 (definite)			
CALLS	2007	Crooks *et al*. [25]			r: 0.41 (c/w MMSE)	0–180
COGTEL	2007	Kliegel *et al*. [26]		r: 0.73		
Rankin’s	2005	Rankin *et al*. [27]		ρ: 0.71–0.89		
Taichman’s	2005	Taichman *et al*. [28]		ICC: 0.54–0.82		
Rapp’s	2012	Rapp *et al*. [29]		r: 0.66		
*Informant-based Tests*						
TDQ	1994	Kawas *et al*. [30]	Sen: 1.00, Spec: 0.9			
IQCODE	1996	Jorm *et al*. [31]	Sen: 0.89, Spec: 0.82	r: 0.91		
SDS	2000	Mundt *et al*. [32]	Sen: 0.90, Spec: 0.86			
*Combined Tests*						
STIDA	1997	Go *et al*. [33]	Sen: 0.93, Spec: 0.92		Weighted Kappa: 0.81 (c/w CDR)	0–81
BSCI	2005	Hill *et al*. [34]			ρ: 0.65 (ADAS) ρ: −0.83 (MMSE)	

Notes: IMCT: Information, Memory, Concentration Test; BRDS: Blessed-Roth Dementia Scale; TICS: Telephone Interview for Cognitive Status; TICS-m: Telephone Interview for Cognitive Status-modified; ALFI-MMSE: Adult Lifestyles and Function Interview-Mini Mental Status Exam; TAMS: Telephone Assessed Mental State; TDQ: Telephone Dementia Questionnaire; TELE: Telephone Screen Protocol; IQCODE: Informant Questionnaire on Cognitive Decline in the Elderly; STIDA: Structured Telephone Interview for the Dementia Assessment; TCAB: Telephone Cognitive Assessment Battery; SDS: Symptoms of Dementia Screener; BSCI: Brief Screen for Cognitive Impairment; COGTEL: Cognitive Telephone Screening Instrument; * NSMD: No significant mean difference.

## 4. Discussion

### 4.1. Limitations of the Existing TBCA Administered via Conventional Telephone

Compared with face-to-face cognitive assessment, the TBCA has taken a big step forward and has many advantages. For example, the cost is lower, assessment time is shorter, it is not restricted by geographical access, and many TBCA instruments were found valid in differentiating people with dementia from the cognitively intact. However, the TBCA instruments identified in this review were all administered by conventional telephone. The conventional telephone, which transmits voices only, may not be technologically adequate to fulfill the cognitive assessment needs for clinical service and research in the near future. 

In the past decade, attention has started to be paid to pre-dementia cognitive impairment. MCI, which is one of the pre-dementia entities, has been found to be associated with elevated rates of conversion to dementia [35,36]. It is arguably considered to be a pre-dementia stage. MCI has heightened the attention of researchers and clinicians in the arena of cognitive assessment because it can be detected at the prodromal stage. Detection of the prodromal stage of dementia may play an important role in advancing the development of pre-dementia interventions. However, MCI cannot be easily identified because people with MCI have no obvious impairment in their daily functioning [35,37]. Their cognitive impairment may be too subtle to be detected by conventional cognitive screening instruments, such as the MMSE [38], which is the instrument from which many of the TBCA instruments were modified (e.g., TICS, TAMS). Although a TBCA was used for MCI screening and was found to be feasible and well received by clients [39], the validity of using the previous TBCA instruments for MCI screening is still questionable. 

Recently, a study [40] found that impairments in specific aspects of executive function may contribute to poor visual learning performance in amnestic MCI (MCI-A), which is a common subtype of MCI in which the primary feature is memory loss, and which is believed to be a prodromal stage of dementia due to Alzheimer’s disease [37]. The intention of the new tests for MCI screening [41] is therefore to assess language by visual learning. This evidence supports that visual communication plays a very important role in cognitive assessment. It is the function lacking in the conventional telephone, and visual tasks are not part of the TBCA instruments administered via the conventional telephone.

Implementing informant-based tests on the conventional telephone resolves the geographical access problem, as the informant does not have to meet the assessor face-to-face. However, the availability problem is not resolved, as both the assessor and the informant have to be available simultaneously to complete the test. It does not allow flexibility for the informant to complete the questionnaire on their own time. File saving and transfer are the functions that are lacking in the conventional telephone.

### 4.2. Recent Developments in Telecommunication and the Penetration of Tele-Technology among the Elderly

A smartphone is essentially a telephone, yet it comprises many features of a computer. It is portable and able to run many programs commonly used on a computer. It incorporates telecommunication features, file saving and transfer, and internet access. It also comprises audio, visual and interactive communication components. In the United States, the percentage of computer and internet use by the elderly increased from 28.4% in 2000 to 55% in 2010 [42]. In Hong Kong, the percentage of personal computer use by the elderly increased from 0.6% in 2000 to 16.8% in 2012 [43]. The percentage of internet use by the elderly increased from 0.2% in 2000 to 15.5% in 2012 [43]. The smartphone user number in Hong Kong increased from 0.63 million in 2005 to 9.38 million in 2013 [44], which is a 14-fold increase in eight years. The smartphone penetration rate was over 50% in the United States in 2010 [45]. This shows that smartphone have many functions superior to the conventional telephone, that they are increasingly gaining popularity, and that the elderly are becoming more used to telecommunication technology. These give the smartphone good potential to be developed for use in TBCAs. However, the impact of the advances in telecommunication upon the methods of cognitive assessment, as well as the current understanding of cognitive impairment as a phenomenon, have yet to catch the attention of clinicians and researchers alike.

### 4.3. Potential Benefits of Using Smartphones in TBCA

The advanced features of smartphones have many potential benefits for TBCA. First, smartphones enable audio, visual and interactive communication. This function allows visual tasks, such as picture naming tasks, to be included in the cognitive assessments. Visual function also enhances communication efficiency, in that instructions can be supplemented by texts or pictures, particularly when the client has some hearing impairment. This feature in the smartphone enables cognitive tasks that previously could only be administered via face-to-face assessment, such as visual memory tests, to be launched on the telephone platform. It also enhances the effectiveness of communication during the assessment. Second, smartphones enable on-screen drawing capture and image transmission. The client can complete drawing tasks directly on the screen according to the instructions given by the assessors. The drawing image can be captured and sent simultaneously to the assessor for immediate analysis. This function allows drawing tasks to be used for cognitive assessment purposes. The assessor can also provide immediate feedback with regard to instructions to clients. Third, smartphones enable computer programs to be run on them, and offer file saving and transfer functions. They allow automated cognitive assessment to be completed without the presence of an assessor. Both subjects and caregivers can complete the testing tasks in an appropriate time and space. The test results and the information provided by the caregivers can be saved and transferred to the assessor without a time limit. This greatly reduces the availability problem among assessors, subjects and caregivers. 

### 4.4. Examples of Use of Smartphones by the Elderly to Manage Their Health

Smartphones have actually been used by the elderly for many other health-related purposes for years. For example, they have been used for the assessment of lifestyle factors for people with dementia [46], for fall risk assessment for the elderly [47], for providing information about dementia [48], for rehabilitation [49], and for disease management [50]. These studies showed that the use of smartphones for health purposes is increasingly gaining in popularity. A recent study [51] even examined the feasibility, reliability and validity of a smartphone-based application to assess cognitive function in the elderly. Apart from classic cognitive tests such as the MMSE and the verbal fluency test, a visual test called the Color-Shape test was included. The study included subjects who were cognitively normal elders with a wide range of experience in smartphone usage, ranging from no previous experience to daily use of a smartphone. This study confirmed that it is feasible to further develop the TBCA among the elderly on the smartphone platform. However, there is a dearth of studies showing that using smartphones for cognitive assessment of the elderly is valid and effective.

### 4.5. The Technological Integration of Smartphones in TBCA and Its Challenges in the Future

There are already many automated cognitive tests available. For example, touch-tone telephones have been used to provide computer-automated dementia screening [52], telephone speech recordings are used to assess cognitive function in the elderly [53], speech-based assessment is used on an automated assessment platform [54]. These studies show that the launch of automated cognitive assessment on the conventional telephone platform is feasible. However, these tests were not rigorously validated to differentiate people with or without cognitive impairment, and they depended mainly on the speech performance of the elderly. Without the integration of visual tests, the applicability of the cognitive assessment in identifying people with cognitive impairment at an early stage is limited. On the internet, there are already many visual and drawing tests, such as Online Clock Drawing Tests [55] and Visual Memory Tests [56]. However, there is a dearth of visual-based automated cognitive assessment that is validated to differentiate people with or without cognitive impairment too. The technology for adapting the automated program to smartphone applications is mature. Automated cognitive assessment can be programmed as a smartphone application. By using smartphones, these tests can be completed by the client at anytime and anywhere not restricted by the availability or locality of the assessor. This smartphone-based cognitive assessment can greatly solve the limitations inherent when the cognitive assessment is delivered via the conventional telephone, such as availability mismatch between assessor and client, audio-only communication, and no visual/drawing test being allowed. This can possibly further reduce the cost of assessment, increase the coverage of the population, and identify cognitive impairment at an earlier stage. 

Although it is practically feasible to enhance TBCA by using smartphones, there are still many challenges before it can be effectively implemented. First, automated cognitive test development is in its infancy, so that there is a dearth of evidence confirming that automated cognitive tests are valid to differentiate people with various levels or types of cognitive impairment. Currently available automated cognitive tests should be validated, or valid smartphone applications for cognitive assessment should be newly developed. Second, readily available automated cognitive assessment without professional input may bear some negative consequences, such as anxiety after misinterpretation of the result. Therefore, a careful follow-up plan should be implemented after the automated cognitive assessment, with adequate consideration of the balance between the rights of the client and the resources of the service providers. Given that the service users can be numerous because of their easy access, a gate-keeping policy against the resources of the service provider should be in place. Third, it is expected that the elderly population will be less familiar with technology when compared with the younger generation [57]. The major factors influencing the intention of the elderly to use smartphones for e-health services are the perceived value and facilitating conditions [58]. For the perceived value, health promotion and education work needs to be done to promote the potential benefits of using smartphones for health purposes. Regarding the facilitating condition, most of the existing smartphones are not designed to accommodate the degenerative changes of the elderly, such as visual and hearing impairment and psychomotor impairment. Pointing is one of the essential psychomotor skills needed to operate the smartphone. Pointing performance is affected by the size of the target on the screen and the feedback after touching the screen [59,60]. Studies have shown that audiotactile feedback condition (*i.e.*, sound and vibration feedback after pressing on the touch screen) enhances the pointing performance of the elderly when operating smartphones [61]. In order to address the possible age-related physical degenerative changes, the screen size and the speaker volume should be increased. Also, an audiotactile feedback condition should be installed. However, the problems faced by the elderly in using and engaging in interactive technology are not limited to physical factors only [62]. It can also be caused by many other factors, such as “too difficult to use” or “looking bad” [63]. Adaptation of smartphones to address factors other than the physical is also important. Technology successfully enhances human-computer interaction by using multimodality. Multimodality means to incorporate multiple input/output methods that are more harmonious to the natural ways of communication for humans, such as speech and gestures. Multimodality successfully causes the elderly to be eager to try new interactive technologies and experience their benefits over their traditional counterparts [64]. Therefore, smartphones should support direct voice input function and speech recognition function to allow the elderly to operate the phone or input text by speaking to the phone only. These measures make smartphones a device more harmonious with natural ways of communicating among the elderly.

With the increasing popularity of automated cognitive assessment and advances in tele-technology, the idea of using smartphones to upgrade the TBCA is made more plausible. However, more research is needed to develop and validate smartphone-based cognitive assessment applications. More research is also needed to promote the potential benefit of using smartphones for health purposes. Finally, the field requires more research in technology development to create facilitating conditions for the elderly to use smartphones.

## 5. Conclusions

TBCA has been developed for more than 20 years and provides access convenience for cognitive and dementia assessment. Previous work on TBCA administered by conventional telephone shows that it is a valid method. However, with advances in the understanding of cognitive impairment, the TBCA administered by conventional telephone may not be adequate to address the cognitive assessment needs in the near future. With advances in telecommunication technology, smartphones have good potential to enhance the applicability of the TBCA by solving these inadequacies. Smartphones demonstrate many advantages that can address the limitations of TBCA administered by conventional telephone, including allowing assessment items based on visual images/drawing, allowing automated cognitive assessment, and allowing file saving and transfer. Smartphones have been successfully used on the elderly for many healthcare purposes, and are expected to increasing gain popularity. Smartphones have also been used for cognitive assessment but there is a dearth of evidence to support their effectiveness. Before smartphones can be successfully used in the TBCA, there are still many challenges, namely the lack of valid automated cognitive assessments available, the projected low intention among the elderly to use smartphones, the lack of facilitating conditions for the elderly to use smartphones, and the lack of adequate support after the TBCA is administered by smartphone. Further research and technological development should focus on the development and validation of smartphone-based cognitive assessment tools/applications, identification of effective measures to enhance the perceived value and facilitating conditions, and identification of a good follow-up plan after the TBCA is administered by smartphone.
